# 小分子抗血管生成药物在非小细胞肺癌中的研究进展

**DOI:** 10.3779/j.issn.1009-3419.2021.102.02

**Published:** 2021-01-20

**Authors:** 妍 窦, 达 姜

**Affiliations:** 050011 石家庄，河北医科大学第四医院肿瘤内科 Department of Oncology, The Fourth Hospital of Hebei Medical University, Shijiazhuang 050011, China

**Keywords:** 抗血管生成抑制剂, 小分子酪氨酸激酶抑制剂, 肺肿瘤, 进展, Angiogenesis inhibitors, Small molecular tyrosine kinase inhibitors, Lung neoplasms, Progress

## Abstract

肺癌是世界上发病率最高的癌症之一，且尚无二线进展后的标准治疗方案，而肿瘤血管生成目前已被确定为恶性肿瘤的重要治疗靶点，小分子多靶点血管激酶抑制剂可通过抑制血管生成相关信号通路，抑制肿瘤血管的生成。目前已开展多项小分子抗血管生成药物治疗非小细胞肺癌的临床试验，且已有部分血管内皮生长因子受体酪氨酸激酶抑制剂（vascular endothelial growth factor receptor-tyrosine kinase inhibitors, VEGFR-TKIs）获批治疗晚期非小细胞肺癌，本文基于国内外多项小分子抗血管生成药物治疗非小细胞肺癌的发展现状，归纳了多个VEGFR-TKIs及成纤维细胞生长因子受体（fibroblast growth factor receptor, FGFR）-TKI单药或联合[包括分别与化疗、表皮生长因子受体（epidermal growth factor receptor, EGFR）-TKIs、免疫治疗、放疗等联合）]治疗非小细胞肺癌的疗效与安全性研究，同时探讨了VEGFR-TKIs可能存在的耐药机制及疗效预测指标等，并对未来抗血管治疗非小细胞肺癌的发展趋势以及存在的潜在问题进行展望，同时为肺癌后续的精准治疗及个体化治疗提供新的思路。

## Abstract (en)

Lung cancer is one of the cancers with the highest incidence in the world, and there is no standard treatment plan after second-line progression. Tumor angiogenesis has now been identified as an important therapeutic target for malignant tumors. Small molecule multi-target vascular kinase inhibitors can inhibit tumor angiogenesis by inhibiting angiogenesis-related signal pathways. At present, a lot of clinical trials of small molecule anti-angiogenic drugs for the treatment of non-small cell lung cancer (NSCLC) have been carried out, and some vascular endothelial growth factor receptor-tyrosine kinase inhibitors (VEGFR-TKIs) have been approved for the treatment of advanced NSCLC. Based on the development status of multiple small molecule anti-angiogenic drugs at home and abroad for the treatment of NSCLC, this article summarizes the efficacy and safety studies of multiple VEGFR-TKIs and fibroblast growth factor receptor (FGFR)-TKI single agents or combination treatments [including combined with chemotherapy, epidermal growth factor receptor (EGFR)-TKIs, immunotherapy, and radiotherapy, *etc*.] for NSCLC, and at the same time discussed the possible existence of VEGFR-TKIs drug resistance mechanisms and efficacy predictors, *etc*., and prospect the future development trend and potential problems of anti-vascular treatment of NSCLC, and provide new ideas for the follow-up precision and individualized treatment of lung cancer.

肺癌是世界上发病率最高的癌症之一，占全球新发癌症的12.9%，致死患者数约占全世界癌症死亡人数的1/5。在我国肺癌的发病率和死亡率均居首位，大约85%的肺癌为非小细胞肺癌（non-small cell lung cancer, NSCLC）^[1]^。随着抗血管生成治疗、靶向治疗及免疫治疗等新手段相继应用于肺癌治疗，不可切除NSCLC的5年生存率有了很大提高，但肺癌仍然是中国最致命的癌症。对于二线或三线治疗失败的患者，很难选择明确的治疗方案，且多数患者不能耐受化疗等相关不良反应，所以肺癌后续治疗的选择仍是目前值得探讨的话题。

自1971年Jodah Folkman教授提出肿瘤新生血管学说以来，肿瘤血管生成已被确定为恶性肿瘤的重要治疗靶标。血管异常对于实体瘤的生长和转移至关重要^[2, 3]^，许多因子和受体与血管生成相关，包括血管内皮生长因子（vascular endothelial growth factor, VEGF）、血小板衍生生长因子（platelet-derived growth factor, PDGF）、成纤维细胞生长因子（fibroblast growth factor, FGF）等。VEGF与血管内皮生成因子受体（vascular endothelial growth factor receptor, VEGFR）结合，诱导VEGFR磷酸化，促进下游信号传导，从而导致肿瘤血管扩展、扭曲和通透性改变。小分子酪氨酸激酶抑制剂（tyrosine kinase inhibitors, TKI），尤其是多靶点血管激酶抑制剂，可选择性抑制下游VEGFR途径介导的激活^[4, 5]^，从而抑制肿瘤血管的生成。目前，各种小分子抗血管生成TKI，例如阿帕替尼、安罗替尼、呋喹替尼和尼达尼布（Nintedanib）等已被应用在许多肺癌临床研究中并进行了评估。与贝伐珠单抗及雷莫卢单抗等大分子单克隆抗体（monoclonal antibodies, mAbs）不同，mAbs特异性强，多与以铂类为基础的化疗相结合，可明显改善总生存期（overall survival, OS），但多数mAbs不可以单独使用。而TKI除了能改善OS，也能改善无进展生存期（progression-free survival, PFS），且可作为单药治疗方案。

## 1 VEGFR-TKIs单药治疗

VEGFR属于酪氨酸激酶（tyrosine kinase, TK）依赖性受体，TKI通过细胞膜进行扩散，竞争细胞内受体酪氨酸激酶结构域ATP结合位点，抑制相应受体的激活，抑制肿瘤血管新生。常见的VEGFR-TKI包括阿帕替尼、安罗替尼、尼达尼布、呋喹替尼、索拉非尼等。

### 1.1 阿帕替尼（Apatinib）

阿帕替尼是VEGFR-2的口服抑制剂，可以通过抑制ABCB1和ABCBG2等外排泵来逆转P-糖蛋白（P-glycoprotein, ABCB1）和乳腺癌耐药蛋白（breast cancer-resistance protein, BCRP/ABCG2）介导的多种耐药，从而降低对常规化疗药物的耐药性^[6]^。2012年中国临床肿瘤学会（Chinese Society of Clinical Oncology, CSCO）发布了阿帕替尼对比安慰剂三线治疗晚期非鳞NSCLC的Ⅱ期随机对照研究，结果显示，阿帕替尼组对比安慰剂组PFS为4.7个月*vs* 1.9个月（HR=0.278, *P* < 0.000, 1）。且客观缓解率（objective response rate, ORR）和疾病控制率（disease control rate, DCR）均显著优于安慰剂组^[7]^。在一项小样本回顾性研究^[8]^中，评估了阿帕替尼对于一线或二线治疗失败的NSCLC患者也可以带来生存获益（PFS为4个月，DCR为61.67%）。也有研究^[9]^表明对于存在*KRAS*突变的晚期肺腺癌患者三线应用阿帕替尼也可获益。阿帕替尼虽在晚期肺癌多线治疗后取得了一定的效果，但阿帕替尼最适用药剂量与疗效间的关系还需要进一步探索。

### 1.2 安罗替尼（Anlotinib）

安罗替尼是一种口服的小分子多靶点TKI，能抑制多种受体酪氨酸激酶[VEGFR1-3、c-KIT、血小板源性生长因子受体α（platelet-derived growth factor receptor α, PDGFR-α）和FGFR1-3]^[10]^。在一项三线及以上应用安罗替尼治疗复发或进展性NSCLC的Ⅱ期随机试验ALTER0302^[11]^中，与安慰剂相比，使用安罗替尼治疗的患者PFS显著获益（4.83个月*vs* 1.23个月，*P* < 0.000, 1）。随后开展的Ⅲ期临床试验ALTER0303^[12]^中，应用安罗替尼对比安慰剂治疗的PFS（5.37个月*vs* 1.40个月，*P* < 0.000, 1）和OS（9.63个月*vs* 6.30个月，*P* < 0.000, 1）都显著延长。亚组分析发现，与表皮生长因子受体（epidermal growth factor receptor, EGFR）野生型的患者相比，具有EGFR突变的患者的OS改善更大^[13]^。基于以上研究成果，安罗替尼于2018年5月8日由国家药品监督管理局（National Medical Products Administration, NMPA）批准用于晚期NSCLC患者的三线治疗。

### 1.3 呋喹替尼（Fruquintinib）

呋喹替尼是新一代小分子VEGFR1-3抑制剂，可抑制VEGF诱导的VEGFR2磷酸化、内皮细胞增殖、体外小管形成和非组织VEGFR2磷酸化^[14, 15]^。一项在国内开展的呋喹替尼治疗晚期非鳞NSCLC的随机Ⅱ期研究（NCT02590965）^[16]^中，单药呋喹替尼组PFS明显优于安慰剂组（3.8个月*vs* 1.1个月，*P* < 0.001），提示呋喹替尼将来有可能成为晚期NSCLC患者标准的三线治疗方案。

### 1.4 索拉非尼（Sorafenib）

索拉非尼是靶向于VEGFR2-3、PDGFRβ、KIT等的多靶点TKI，一项治疗NSCLC的随机安慰剂对照Ⅲ期试验（MISSION）^[17]^中，索拉非尼作为三线/四线治疗尽管PFS稍增加（2.8个月*vs* 1.4个月，*P* < 0.000, 1），但不能显著增加患者的OS（8.2个月*vs* 8.3个月，*P*=0.47）。虽然索拉非尼单药用于NSCLC的试验结果不尽人意，但后续索拉非尼与化疗联合治疗NSCLC的试验数据仍取得不错结果，仍可以期待后续联合治疗后的进展。

### 1.5 Linifanib

Linifanib（ABT-869）是三磷酸腺苷竞争性TKI，可选择性抑制VEGF（FLT1、KDR、FLT4）和PDGF（PDGFRα、CSF-1R、KIT、FLT3）受体^[18]^。临床前研究^[19]^表明，在多种肿瘤模型中Linifanib均可增强卡铂和紫杉醇的活性。一项Linifanib/安慰剂联合卡铂+紫杉醇治疗晚期非鳞NSCLC的随机Ⅱ期研究中，Linifanib组中位PFS明显优于安慰剂组（安慰剂组：5.4个月，95%CI：4.2个月-5.7个月；Linifanib 7.5 mg组：8.3个月，95%CI：4.2-10.8，Linifanib 12.5 mg组：7.3个月，95%CI：4.6-10.8）^[20]^。由此可见在晚期非鳞NSCLC患者治疗中加入Linifanib也不失为一种选择。

## 2 FGFR-TKIs单药治疗

Dovitinib（TKI258）是靶向于VEGFR1-3、FGFR1-3、PDGFR和KIT等的多靶点TKI^[21]^。一项单臂Ⅱ期临床试验^[22]^研究Dovitinib治疗具有FGFR扩增的晚期鳞状NSCLC患者，Dovitinib给药的中位持续时间达2.5个月，ORR为11.5%，DCR达50%，中位OS为5.0个月，中位PFS为2.9个月，可见FGFR-TKI亦为鳞状NSCLC日后进一步治疗方案提供新的思路。

## 3 VEGFR-TKIs联合治疗

与传统化疗和EGFR-TKI不同，VEGFR-TKIs可作用于肿瘤微环境（tumor microenvironment, TME）以使现有的肿瘤血管退化并抑制肿瘤的新生血管生成。多项临床研究表明，抗血管生成治疗与其他肺癌局部或全身性治疗联合使用，包括化疗、小分子靶向治疗、免疫治疗、放疗等，具有更好的抗肿瘤作用并延缓耐药的发生。

### 3.1 VEGFR-TKIs联合化疗

VEGFR-TKIs使存活的肿瘤血管正常化^[23]^，推动化疗药物向肿瘤组织内运输，从而提高化疗效果^[24]^。尼达尼布（Nintedanib）是一种小分子TKI，可抑制多种受体酪氨酸激酶：VEGFR1-3、FGFR1-3、PDGFR-α/-β、RET、FMS样酪氨酸激酶3（FMS-like tyrosine kinase 3, FLT3）和Src家族酪氨酸蛋白激酶（Src family protein tyrosine kinases, SrcPTKs）。通过竞争性结合上述胞内受体激酶结构域上的三磷酸腺苷位点，阻滞胞内信号传导，抑制成纤维细胞的增殖、迁移和转化。一项随机双盲Ⅲ期临床试验LUME-Lung 1^[25]^评估了多西他赛联合尼达尼布二线治疗晚期NSCLC，可显著延长患者PFS（3.4个月*vs* 2.7个月），且在肺腺癌亚组中，联合方案可使患者OS延长2.3个月。2014年11月，欧盟批准将尼达尼布联合多西他赛用于晚期或转移性肺腺癌的二线治疗。

### 3.2 VEGFR-TKI联合EGFR-TKI治疗

VEGF和EGFR共有许多重叠和平行的下游途径。多种血管生成生长因子通过EGFR信号调节而增加，包括VEGF、白介素8（interleukin-8, IL-8）和碱性成纤维细胞生长因子（basic fibroblast growth factor, bFGF），可通过阻断VEGF使EGFR自分泌信号传导下调，从而抑制肿瘤生长^[26]^。临床前研究^[27]^发现，在胃癌小鼠模型中DC101（VEGFR2抗体）和C225（EGFR抗体）的联合使用可通过减少肿瘤血管和增加内皮细胞凋亡来抑制肿瘤细胞的增殖。阻断VEGFR信号通路可逆转在EGFR-TKIs治疗中由T790M突变引起的耐药^[28]^。舒尼替尼（Sunitinib）是靶向于VEGFR1-3、PDGFR-α/-β、c-KIT、FLT3、集落刺激因子1受体（colony stimulating factor 1 receptor, CSF1R）和RET的TKI。在一项舒尼替尼联合厄洛替尼对比厄洛替尼单药治疗既往治疗失败未进行EGFR基因检测的NSCLC患者的Ⅲ期试验^[29]^中，舒尼替尼联合组显著增加了患者PFS（3.6个月*vs* 2.0个月）和RR（10.6% *vs* 6.9%），但OS没有明显差异（9.0个月*vs* 8.5个月）。现在仍有多项试验正在进行中，其中Ahead-L303研究-阿帕替尼联合吉非替尼一线治疗晚期EGFR突变的非鳞NSCLC患者，有望获得阳性结果。另外一项安罗替尼联合厄洛替尼一线治疗EGFR突变阳性晚期NSCLC患者的临床研究，PFS结果仍在跟进中，值得期待。

### 3.3 VEGFR-TKI联合免疫检查点抑制剂治疗

VEGF可诱导肿瘤相关的免疫缺陷，并在肿瘤细胞规避免疫监视的免疫抑制微环境中发挥重要的负性调节作用。一方面，VEGF抑制淋巴细胞的黏附以激活内皮细胞，同时抑制淋巴细胞通过内皮细胞进入肿瘤的转运，从而阻止T细胞浸润^[30]^。VEGF还可通过作用于Fas配体抑制T细胞动员和运输^[31]^。另一方面，VEGF通过多种机制发挥免疫抑制作用，包括诱导调节性T细胞（Tregs）增殖和抑制树突状细胞（dendritic cell, DC）成熟^[32]^。VEGFR-TKI可以通过降低肿瘤的密度缓解血管压力，使异常的血管系统正常化，增加免疫效应细胞的渗透，并将肿瘤固有的免疫的抑制性微环境转化为免疫支持的微环境^[33]^。2019年美国临床肿瘤学会（American Society of Clinical Oncology, ASCO）发表一项SHR-1210联合阿帕替尼用于三线治疗失败的晚期非鳞NSCLC的Ib期试验^[34]^，结果表明，程序性死亡受体1（programmed cell death protein 1, PD-1）联合阿帕替尼在晚期NSCLC患者治疗中表现出良好的抗肿瘤疗效，ORR为30.8%，DCR为92.8%，中位PFS达到24周。2019年世界肺癌大会（World Conference on Lung Cancer, WCLC）上报道了一项信迪利单抗（Sintilimab）和安罗替尼联合一线治疗晚期NSCLC的结果，ORR（72.7%）已达到主要终点，DCR为100%^[35]^。初步结果显示，对于晚期NSCLC患者，免疫检查点抑制剂（immune checkpoint inhibitors, ICIs）与抗血管生成剂联合使用具有可观的抗肿瘤活性，但在真实世界中应用需更多临床数据支持。

### 3.4 VEGFR-TKI联合放射治疗

长程放疗会增加血管内皮生长因子表达，使肿瘤产生放射抵抗，影响疗效，而VEGFR-TKI可下调血管内皮生长因子核糖核酸及蛋白的表达，发挥放疗增敏的作用。一项安罗替尼联合放疗治疗NSCLC伴脑转移患者的小样本（40例）临床分析^[36]^表明，安罗替尼联合放疗组ORR及DCR分别为60.00%和90.00%，单纯放疗组为35.55%和55.00%（*P* < 0.05）。同时有研究^[37]^表明，安罗替尼辅助脑放疗，通过时机的选择可提高NSCLC脑转移患者的疗效并延长患者生存期。

## 4 VEGFR-TKIs相关不良反应

多数小分子抗血管生成药物常见不良反应包括高血压、皮疹、出血、手足综合征、乏力及相关肝肾毒性等，不同VEGFR-TKI相关不良反应（adverse event, AE）略有差异，同时与化疗、靶向及免疫治疗等联合治疗时，AE也各有不同。

### 4.1 VEGFR-TKIs单药治疗相关AE

在ALTER0303研究中^[12]^，应用安罗替尼单药咯血发生率为20.4%，3级AE发生率为3.1%，未发现严重出血及治疗相关AE导致的死亡。不同药物的AE的侧重点亦不同，且VEGFR-TKI用药剂量与AE发生率及疗效之间的关系仍需后续进一步临床试验明确。

### 4.2 VEGFR-TKIs联合治疗相关AE

在LUME-Lung 2^[38]^研究中，与安慰剂组相比，尼达尼布联合培美曲塞组的常见的3级及以上AE为肝功能损伤及腹泻发生率增加，但高血压、出血等风险没有差异。一项研究安罗替尼联合厄洛替尼一线NSCLC治疗的小样本临床试验^[39]^中，皮疹（17.24%）的3级不良事件发生率很高，同时在所有患者中均观察到1级-4级高血压。一项信迪利单抗和联合安罗替尼一线治疗晚期NSCLC的试验^[35]^中，最常见的AE是高血压，≥3级治疗相关AE的发生率为27.3%。由此可见，当VEGFR-TKI与其他治疗方式联合时，AE类型也不尽相同，如何抉择具体联合的方式及联合剂量，仍需未来进一步研究。

## 5 VEGFR-TKIs可能存在的耐药机制

肿瘤微环境中的各组成成分可通过多种途径调节肿瘤新血管生成，促使肿瘤的发展和转移。VEGFR-TKI不仅可以清除肿瘤生长和转移所必需的血管，还可以调节肿瘤免疫微环境。目前VEGFR-TKI可能存在的耐药机制主要由肿瘤微环境反应性变化引起，其中包括其他促血管生成因子的表达、缺氧加重及肿瘤细胞自噬等。

### 5.1 其他促血管生成因子的表达

VEGF是参与血管生成的主要因子，能刺激血管内皮细胞增殖，促进体内新血管生成。胎盘生长因子（placenta growth factor, PLGF）、FGF、PDGF、肿瘤坏死因子-α（tumor necrosis factor-α, TNF-α）等其他促血管生成因子可通过与内皮细胞或其上的受体作用促进内皮细胞的迁移、增殖等活性^[40]^。当药物阻断VEGF/VEGFR参与的信号通路时，其他血管生长因子介导的信号通路可能被激活，从而导致肿瘤复发^[41]^。多种血管生成信号传导途径的激活可导致肿瘤细胞抵抗抗血管生成疗法。

### 5.2 肿瘤缺氧环境

缺氧可能导致血管重构（vascular remodeling, VR），重构的中心血管管腔直径增大，血管壁细胞增殖，ephrinB2等因子表达增加，重构的血管的稳定性增加，重建肿瘤血运^[42, 43]^。同时肿瘤微环境缺氧区域的增加，会增加肿瘤干细胞数量，有助于克服缺氧所致的营养不良，并通过增殖、侵袭、扩散、转移等途径逃避恶劣的微环境，产生耐药。缺氧诱导因子（hypoxia inducible factors, HIFs）是一类氧依赖转录激活因子^[44]^，可促使肿瘤细胞适应缺氧环境，有研究^[45]^表明其可调控与肿瘤细胞侵袭和转移相关的基因。缺氧与蛋白激酶B（serine/threonine protein kinase, Akt）活性之间存在一定的联系。相关研究表明，在缺氧的细胞中Akt被激活，从而促进细胞生存和肿瘤发生；这些均为抑制肿瘤耐药提供了线索。

## 6 VEGFR-TKI可能相关的疗效预测指标

### 6.1 液体活检预测指标

ALTER0303试验^[46]^确定了激活的循环内皮细胞（activated circulating vascular endothelial cells, aCECs）是安罗替尼治疗期间预测PFS的潜在生物标志物。同时一项基于该实验试验患者循环DNA的研究^[47]^确立了3个预测指标：胚系和体系细胞突变负荷（G+S MB）、非同义和同义突变负荷（N+S MB）、循环DNA的不利突变评分（unfavorable mutation scale, UMS）。并且建立了预测模型，即肿瘤突变指数（tumor mutation index, TMI），结果显示：G+S MB及N+S MB较低患者对安罗替尼的敏感性均高于突变负荷高的患者；TMI越低越可能从安罗替尼中获益。且ARID1A和BRCA2的获得性突变可能与安罗替尼获得性耐药性相关。同时存在IDH1外显子4突变可能使安罗替尼治疗的获益降低。一项临床前研究^[48]^发现microRNA-6077可通过抑制葡萄糖转运蛋白1（glucose transporter 1, GLUT1）表达，增强肺腺癌细胞对安罗替尼的敏感性。

### 6.2 影像学预测指标

Wang等^[49]^发现在抗血管生成治疗时血容积（blood volume, BV）往往比普通CT征象早1个月-2个月出现变化，与aCECs结合还可提高预测疗效的敏感与可靠性。因此一些肿瘤内血流灌注指标也可以作为疗效预测指标。目前，VEGFR-TKI疗效相关生物标志物的研究还在进行中，探寻精准预测疗效及预后的生物标志物，为抗血管靶向治疗的精准应用提供指导。

## 7 展望

抗血管靶向治疗目前疗效可观，为肺癌患者的进一步治疗提供更多的选择，但目前仍存在一些亟待解决的问题：①小分子抗血管生成药物副作用相对较小，患者耐受好，其一线、二线的联合治疗模式是否能够使患者更加获益、联合治疗模式中最合适的剂量和给药时序等问题有待进一步临床试验验证；②多种血管生成信号传导途径的激活可导致肿瘤细胞抵抗抗血管生成疗法，造成单靶点药物耐药。因此克服耐药的关键在于明确“旁路激活”和多靶点的同时抑制，探索明确耐药原因及克服耐药的方法还需要进一步努力；③NSCLC中与抗血管生成药物疗效有关的生物标志物的探索仍处于起步阶段，因此如何筛选抗血管治疗优势人群、实现精准治疗也是后续需要研究的重点。或许，一些肿瘤内血流灌注指标也可以作为疗效预测指标；④抗血管生成药物对作用血管的选择性欠佳，对于机体正常血管也存在一定损伤，开发特异性针对肿瘤血管及血管生成驱动基因的药物或许是抗血管生成药物未来研发的方向。
